# Cooperativity and interaction energy threshold effects in recognition of the −10 promoter element by bacterial RNA polymerase

**DOI:** 10.1093/nar/gkt541

**Published:** 2013-06-14

**Authors:** Vladimir Mekler, Konstantin Severinov

**Affiliations:** ^1^Department of Molecular Biology and Biochemistry, Waksman Institute of Microbiology Rutgers, State University of New Jersey, Piscataway, NJ 08854, USA and ^2^Institutes of Molecular Genetics and Gene Biology, Russian Academy of Sciences, Moscow 119334, Russia

## Abstract

RNA polymerase (RNAP) melts promoter DNA to form transcription-competent open promoter complex (RP_o_). Interaction of the RNAP σ subunit with non-template strand bases of a conserved −10 element (consensus sequence T_−12_A_−11_T_−10_A_−9_A_−8_T_−7_) is an important source of energy-driving localized promoter melting. Here, we used an RNAP molecular beacon assay to investigate interdependencies of RNAP interactions with −10 element nucleotides. The results reveal a strong cooperation between RNAP interactions with individual −10 element non-template strand nucleotides and indicate that recognition of the −10 element bases occurs only when free energy of the overall RNAP −10 element binding reaches a certain threshold level. The threshold-like mode of the −10 element recognition may be related to the energetic cost of attaining a conformation of the −10 element that is recognizable by RNAP. The RNAP interaction with T/A_−12_ base pair was found to be strongly stimulated by RNAP interactions with other −10 element bases and with promoter spacer between the −10 and −35 promoter elements. The data also indicate that unmelted −10 promoter element can impair RNAP interactions with promoter DNA upstream of the −11 position. We suggest that cooperativity and threshold effects are important factors guiding the dynamics and selectivity of RP_o_ formation.

## INTRODUCTION

Formation of the transcription-competent open promoter complex (RP_o_) by bacterial DNA-dependent RNA polymerase (RNAP) is a critical checkpoint on the pathway of gene expression. In RP_o_, the DNA duplex is melted over a stretch of 12–15 bp, which makes the transcription start point (position +1) accessible to the RNAP catalytic center. RNAP initiates transcription in the form of a holoenzyme (subunit composition αIαIIββ’ωσ). The dissociable specificity subunit σ is required for both promoter recognition and melting (1,2). Specific interactions of the *Escherichia coli* primary σ subunit (σ^70^) with non-template strand (nt-strand) nucleotides of conserved −10 promoter element (consensus sequence T_−__12_A_−__11_T_−__10_A_−__9_A_−__8_T_−__7_) are an important source of energy-driving localized melting of σ^70^-dependent promoters (3–7). The strand separation usually is initiated at the −11A/T base pair and propagates in the downstream direction (2). The −12 bp likely remains in the double-stranded (ds) form in most promoters (8,9). At physiological conditions, the RP_o_ formation is a highly cooperative process (2,10–12). Yet, intermediate promoter complexes with transcription bubbles not extended to include the transcription start point have been detected at several promoters (12–14) as well as when studying RNAP mutants (15,16). In contrast, promoter complexes bearing partially melted −10 promoter element have not been revealed at physiological temperatures, implying a particularly high degree of cooperativity in unpairing of the −10 element bases.

Oligonucleotides and fork junction promoter fragments containing single-stranded (ss) extensions corresponding to the nt-strand of the −10 promoter element have been used as model substrates to study RNAP interactions with melted DNA in promoter complexes (3,4). Binding studies using these DNA probes confirmed that in the context of the RNAP holoenzyme, σ subunit recognizes the −10-nt-strand sequence in the single-stranded form (3–6,9,17). Recently reported structures of σ domain 2 and RNAP holoenzyme complexes with model promoter fragments reveal that the nt-strand bases of consensus −10 element interact with numerous residues from σ conserved regions 2 and 1.2 with multiple σ residues simultaneously contacting more than one nucleotide (18,19). In the structures, the nt-strand segment of the −10 element adopts a conformation that is incompatible with the ds DNA conformation, particularly because the A-11 and T-7 bases are flipped out of the DNA base stack (18,19). These results indicate that the recognition of the −10 element must be coupled with its unwinding and melting.

The molecular details of events that trigger the −10 promoter element recognition and strand separation remain unclear. In the light of the proposed mechanism of the −10 element recognition, we reasoned that studying interdependences between RNAP interactions with individual −10 element nucleotides may help clarify fine details of these processes. Although non-additive effects of multiple substitutions in −10 element bases on transcription (20) and RNAP binding (5) have been observed, experimental data on interdependences between partial σ interactions with the −10 element bases are lacking. Here, we systematically studied mutual effects of partial RNAP interactions with −10 element bases in the context of RNAP complexes with model promoter fragments by using a highly sensitive and quantitative fluorometric RNAP molecular beacon assay. The data reveal a strong degree of cooperation between specific RNAP contacts with individual −10 element nucleotides and show that the recognition of the −10 element bases occurs only when the overall interaction acquires a free energy below a ∼−3 kcal/mol threshold. We suggest that the threshold effect contributes to the selectivity of open promoter complex formation by hindering RNAP binding to non-promoter DNA.

## MATERIALS AND METHODS

### Proteins

*Escherichia coli* RNAP core was purchased from Epicenter. RNA polymerase holoenzyme containing the σ^70^ derivative labeled at position 211 with fluorescent label 5-tetramethylrhodamine (RNAP beacon) was prepared as previously described (6).

### DNA probes

DNA oligonucleotides were synthesized by Integrated DNA Technologies. Fork junction and double-stranded DNA probes were prepared as described previously (6).

### Fluorometric assays

Fluorescence measurements were performed using a QuantaMaster QM4 spectrofluorometer (PTI) in transcription buffer [40 mM Tris–HCl (pH 8.0), 100 mM NaCl, 5% glycerol, 1 mM DTT and 10 mM MgCl_2_] containing 0.02% Tween 20 at 25°C. Final assay mixtures (800 μl) contained 1 nM labeled RNAP holoenzyme and DNA probes at various concentrations. The fluorescence intensities were recorded with an excitation wavelength of 550 nm and an emission wavelength of 578 nm.

To obtain equilibrium dissociation constants (*K*_d_), the experimental dependence of the fluorescent signal amplitude (F) on DNA probe concentration was fit to Equation (1), unless otherwise noted (6,21).
(1)
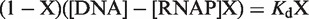

where X = (F − F_0_)/(F_max_ − F_0_), F_0_ is the initial value of the amplitude, and F_max_ is the limiting value of the amplitude at [DNA] = ∞. The data were analyzed using SigmaPlot software (SPSS, Inc.). The experimental variation of F/F_0_ among replicate measurements usually did not exceed 10% of the average value. The *K*_d_ values presented are averages obtained from two to three individual experiments, the error is 20% for *K*_d_ > 0.4 nM and ∼50% for *K*_d_ within the range of 0.2–0.4 nM.

An equilibrium competition-binding assay was used to measure affinity of tight *E.coli* RNAP complexes (*K*_d_ < 0.2 nM), representative experimental data are shown in Supplementary Figures S2B and S3B. A double-stranded [−58/−14] probe (shown in Supplementary Figure S2A) producing negligible signal on binding to the RNAP beacon was used as a reference competitor, as described previously (6). Time-dependent fluorescence changes were monitored after manual mixing of RNAP beacon (800 μl) and a DNA probe (<20 μl) in a cuvette; the mixing dead-time was 15 s.

In line with previous works, we used values of the free energy gain/loss resulted from substitution a consensus base Y for a non-consensus base Z at position N in the −10 element ΔΔG(Y_N_Z) to characterize strengths of the specific interactions in RNAP complexes with studied templates. The changes in ΔG were calculated using Equation (2):
(2)
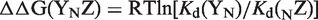



**Figure 1.**
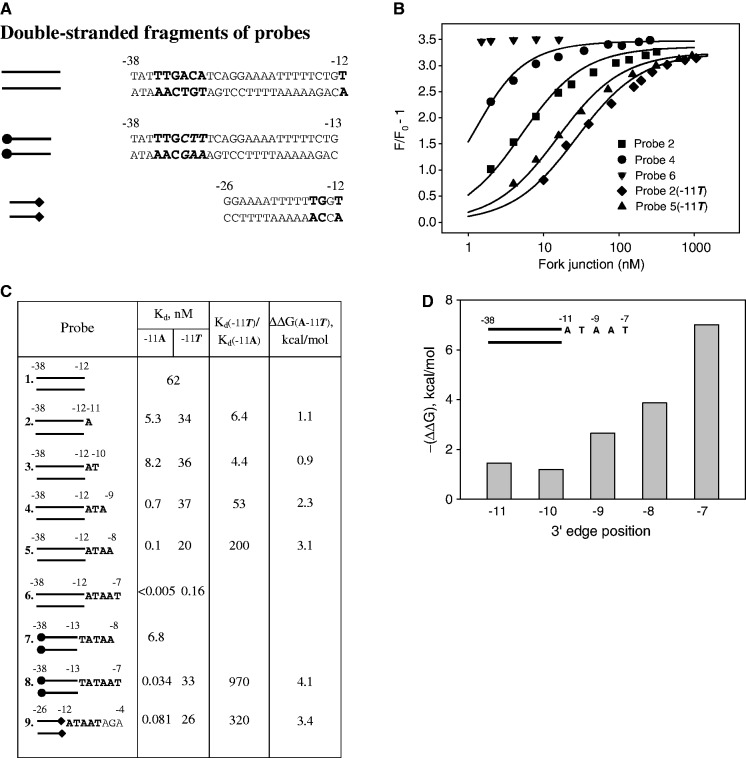
Effect of the A−11T substitution on RNAP binding to promoter fragment probes. (**A**) Sequences of double-stranded parts of fork junction probes. Non-consensus −35 and −10 element bases are shown in italic. (**B**) Representative experimental data on titration of the RNAP beacon with fork junction probes. Continuous lines correspond to non-linear regression fit of the data. (**C**) The panel shows structures of fork junction probes, *K*_d_ for the RNAP-probe binding, and free energy losses because of the A−11T mutation. (**D**) Calculated free energies of RNAP interactions with consecutive extensions of nt-strand segment in fork junction probes.

Free energy gain resulted from RNAP binding to the single-stranded segments of fork junctions 2–5 (the structures are shown in Figure 1C and Supplementary Figure S1) was calculated using Equation (3)
(3)
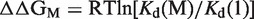

where *K*_d_(M) and *K*_d_(1) are dissociation constants for assayed probes and probe 1 that corresponds to the double-stranded fragment of the probes. As *K*_d_(6) could not be directly measured (see later in the text), ΔΔG_6_ was evaluated using Equation (4)
(4)
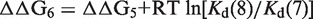

assuming that improvements of fork junction affinities because of extension of their ss segments from −8 to −7 are equal in the context of probes 5, 6 and 7, 8.

## RESULTS

### DNA probes

To discern interdependencies of partial RNAP interactions with individual nucleotides of the −10 promoter element, we measured RNAP affinity to a large set of model promoter fragments. The structures of DNA probes used are presented in Supplementary Figure S1 and are also schematically depicted in main figures. The majority of probes are based on T5 N25, a strong promoter containing consensus −10 element. Some experiments were performed with probes based on the sequence of a weak Pr promoter of *Pseudomonas putida* bearing a suboptimal −10 element (22). The affinities of DNA probes to RNAP were characterized by dissociation constants of their complexes with RNAP as determined by the fluorometric RNAP molecular beacon assay (6). The *K*_d_ values varied widely—over a 4-log range. With its high sensitivity and low intensity of non-specific background signal, the RNAP beacon assay is ideally suited for performing such measurements, as it allows to quantitatively measure both weak and strong interactions.

### Binding of fork junction promoter fragments reveals strong cooperativity of individual −10 element nt-strand nucleotides interactions with RNAP

An adenine at the −11 position and a thymine at the −7 position are the most conserved bases of the −10 element (23). Introduction of non-consensus bases at the −11 position generally strongly decreases promoter activity (24,25). To test the effects of specific RNAP contact with −11A on RNAP interactions with other −10 element nt-strand nucleotides, we compared RNAP affinities with a series of fork junction DNA probes bearing consecutive one-nucleotide extensions of the nt-strand from the −11 to −7 positions (Figure 1A and C) and to a set of similar probes in which the −11 position was occupied by a non-consensus T. Probes 1–6 bear consensus −35 element sequence TTGACA. The *K*_d_ of RNAP complexes with each probe, the ratios of *K*_d_ values for matching probes bearing either an A or a T at the −11 position and corresponding free energy changes caused by the A-11T substitution are shown in Figure 1C; Figure 1B shows representative experimental data. *K*_d_ for RNAP complex with fork junction 6 (bears consensus −10 element) could not be calculated from data shown in Figure 1B, as RNAP binding to this probe was too strong and fluorescence intensity reached saturation level at a minimal probe concentration used. Determination of the *K*_d_ value by equilibrium competition-binding assay also could not be carried out, as reactions did not reach equilibrium even after a 20-h incubation (data not shown), which can be explained by slow dissociation of RNAP complex with this probe (26). Therefore, we determined a change in *K*_d_ caused by the extension from −8 to −7 in the context of fork junctions 7 and 8 (Figure 1C and Supplementary Figure S2), whose affinity to RNAP is weakened because of a non-consensus −35 element sequence TTGCTT (a −35 element of the T5 N25 promoter) and a junction point at position −13 rather than at −12 as in probes 1–6 (4).

The calculated free energies of RNAP binding to consensus single-stranded segments of fork junctions are shown in Figure 1D. Overall, the data presented in Figure 1C and 1D show that for the most part, consecutive one-nucleotide extensions considerably improve affinities of −11A containing probes. The only exception is probe 3, where the introduction of −10T causes a drop in affinity compared with the shorter probe 2. A similar inhibitory effect of an extra top strand −10 nucleotide on formation of heparin-resistant RNAP-fork junction complexes was reported by Guo and Gralla (4). In contrast to the −11A containing probes, for the −11T series of probes only the extension from −8 to −7 resulted in a large increase in affinity, whereas other one-nucleotide extensions had at most a slight effect on binding. The A−11T substitution caused an ∼5-fold drop in affinities of probes with 3′ termini at −11 and −10. This ratio increased to ∼200 for probes extending to −8, whereas a nearly 1000-fold difference was observed in the context of probes extended to −7. The A−11T substitution also caused a large ∼300-fold effect on RNAP binding to fork junction 9 that does not contain the −35 element but bears an extended −10 element and includes the −6 to −4 bases that interact with the σ conserved region 1.2 (19,27,28). Clearly, these results indicate that efficiency of −11A recognition is strongly stimulated by RNAP contacts with other −10 element bases.

**Figure 2.**
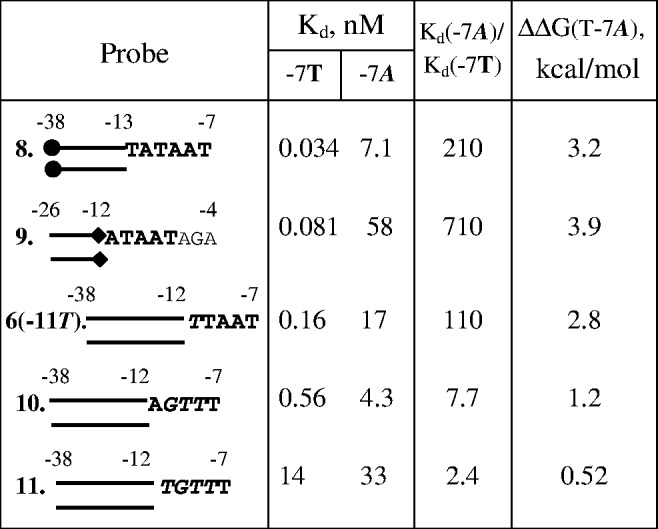
Effect of the T−7A substitution on RNAP binding to fork junction probes. The sequences of double-stranded parts of the probes are shown in Figure 1A. Non-consensus −10 element bases are shown in italic.

The fact that the introduction of −7T confers a ∼120-fold improvement in affinity of −11T probes [compare *K*_d_ values for probes 5(−11T) and 6(−11T) in Figure 1C] demonstrates that specific interaction of −11A with RNAP is not strictly required for the recognition of −7T in the context of fork junctions. We evaluated the specificity of RNAP interaction with −7T in the context of several fork junctions with non-consensus bases at positions −11 to −8 (Figure 2). Introduction of A at position −7 caused, respectively, 210- and 710-fold drops in affinity of probes 8 and 9 with consensus −10 element. A 110-fold drop was observed in the context of a probe with a non-consensus -11T [probe 6(−11T)]. A much lower, ∼8-fold, effect was observed with probe 10 bearing a G_−__10_T_−__9_T_−__8_ non-consensus base stretch. Finally, the T−7A substitution caused only a 2.4-fold effect in the context of fork junction 11 with non-consensus bases at positions −11 to −8. Thus, the efficiency of −7T recognition is clearly modulated by the strength of RNAP contacts with other −10 element bases.

Overall, the results show that specific interactions between individual −10 element bases and RNAP are highly interdependent. Further, a strong specific interaction between RNAP and −10 promoter element bases occurs only when free energy of the overall RNAP interaction with the −10 element reaches a certain critical level. The specific RNAP interaction with −11A is much weaker in fork junctions with short ss extensions (probes 2 and 3) than in fork junctions 8 and 9 bearing ss extensions spanning the entire −10 element [ΔΔG(A−11T) ∼1 and ∼4 kcal/mol, respectively]. The threshold effect is also pronounced for less conserved −10T, −9A and −8A bases. Indeed, the T_−__10_A_−__9_A_−__8_ segment improved the binding of probe 5 as compared with probe 2 ∼50-fold, whereas in the context of −11T substituted probes, this segment increased the binding only ∼2-fold (Figure 1C). A similar effect is observed for −7T base recognition in fork junctions 8, 9 and 11 bearing all-consensus (probes 8 and 9) or non-consensus (probe 11) bases within the −11 to −8 segment (Figure 2). These results may be explained by individual interactions between RNAP and −10 element nt-strand nucleotides cooperatively contributing to retention of a conformation of the −11 to −7 segment backbone (18,19) that favors recognition of the −10 element bases. We suggest that the ∼3 kcal/mol difference in ΔΔG(A−11T) values for probes 2, 3 and 8, 9 approximately corresponds to the energetic cost required to retain such conformation. The threshold energy value likely depends on promoter sequence, in particular on identity of the −12 bp (see later in the text).

### Inhibition of fork junction DNA binding by a terminal nt-strand −10 base

**Figure 3.**
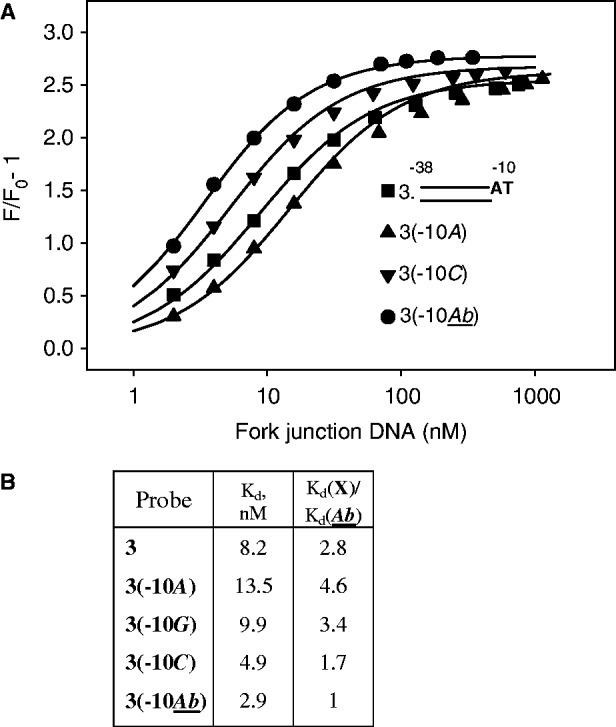
Effect of substitutions at the −10 position on RNAP binding to derivatives of fork junction 3. (**A**) Structures of DNA probes and titration of the RNAP beacon with fork junction probes. Continuous lines correspond to non-linear regression fit of the data. The sequence of double-stranded part of the probes is shown in Figure 1A. *Ab* in the structure of probe 3(−10*Ab*) stands for abasic substitution. (**B**) Calculated *K*_d_ values.

We further examined inhibition of RNAP binding observed on the introduction of consensus −10 T (probe 3) into probe 2 (Figure 1C). We determined *K*_d_ values for RNAP complexes with derivatives of probe 3 bearing an A, a G, a C or an abasic site at the −10 position (shown in Figure 3A) and found that only the abasic probe [probe 3(−10*Ab*)] bound slightly better than probe 2. The *K*_d_ values for other probes were higher than that of probe 3(−10*Ab*) by 1.7- to 4.6-fold (Figure 3A and B). This result correlates with reported negligible effect of the introduction of an abasic site at the −10 position on heparin resistance of an RNAP-fork junction complex (29). It seems that the need to unstack the −11A base, which is a prerequisite for its specific binding by σ (18,19), may, at least in part, account for the slight energetically unfavorable effect of the presence of a base at position −10 observed in fork junction 3 and its derivatives. Disruption of stacking interaction between the −11 and −10 bases should consume a part of the binding energy, whereas the contribution of the −10 position interaction with RNAP to the overall binding energy is likely to be insignificant, as RNAP interacts only with the backbone of the −10 nt in reported structures of RNAP complexes with promoter fragments (18,19). Consistent with the latter suggestion, substitutions of −10T have a rather slight effect on the affinity of an oligonucleotide probe 30 corresponding to positions −12 to +2 of the nt-strand segment of the T5 N25 promoter (Supplementary Table S1). It is noteworthy also that purines at −10 cause somewhat higher inhibitory effects than pyrimidines (Figure 3B). This is consistent with the expectation that stacking interactions between neighboring purines should be stronger than between a purine and a pyrimidine because of higher surface area of the contact (30).

### Recognition of −7T base in the context of fork junctions based on the sequence of Pr promoter

**Figure 4.**
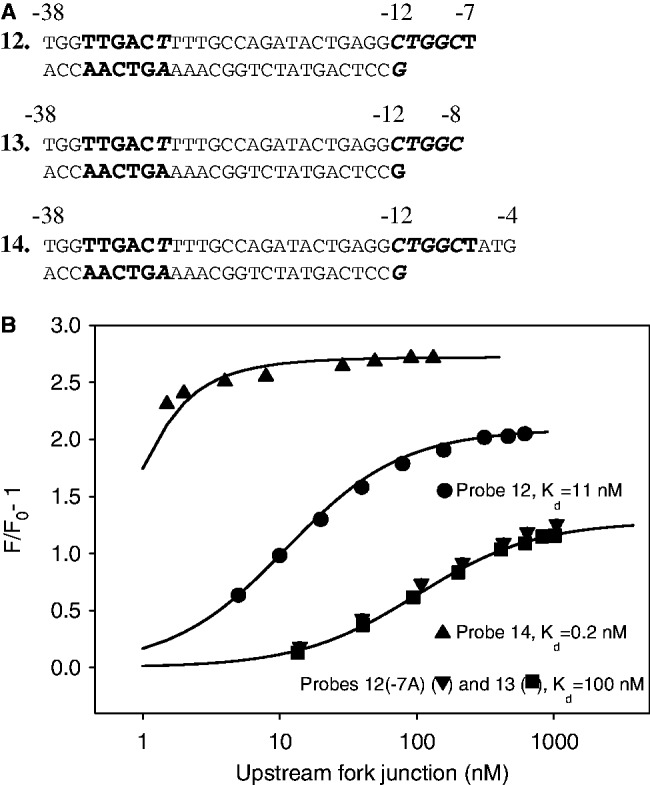
RNAP beacon-binding assay for fork junctions based on the sequence of Pr promoter bearing a suboptimal −10 element. (**A**) Structures of a fork junction probes 12, 13 and 14 based on the Pr promoter sequence and calculated *K*_d_ values. Non-consensus −35 and −10 element bases are shown in italic. (**B**) Titration of the RNAP beacon with fork junction probes. Continuous lines correspond to non-linear regression fit of the data.

The σ^70^-dependent Pr promoter controls catabolism of phenolic compounds by *P. putida* CF600 (22). The Pr promoter bears a poor −10 element C_−__12_TGGCT_−__7_ containing only one consensus base −7T (22). Consequently, the Pr promoter is intrinsically weak and requires ppGpp and DksA for optimal activity (31). Substitutions of −7T for any other base abolish activity (31). We wondered whether the critical importance of −7T for Pr activity is displayed in the context of RNAP interaction with fork junction probes. We measured RNAP binding to Pr-based fork junction probe 12 and to control probes that either bore an A at the −7 position [probe 12(−7A)] or lacked the −7 nucleotide altogether (probe 13) (Figure 4A). The *K*_d_ for probe 12 was 11 nM, whereas the *K*_d_ values for control 12(−7A) or 13 probes were found to be ∼100 nM (Figure 4B). This result demonstrates that RNAP clearly recognizes −7T in fork junctions based on the Pr promoter, whereas the RNAP interaction with −7T in probe 12 is much weaker than in probe 8 bearing consensus −10 element bases [ΔΔG(T-7A) values of 1.3 and 3.2 kcal/mol, respectively]. Further extending the nt-strand from −7 to −4 in probe 14 considerably increased the binding affinity compared with probe 12 (Figure 4B), indicating that the RNAP interaction with the Pr discriminator segment is not affected in the probe.

### Recognition of the T/A_−__12_ base pair depends on RNAP interactions with other −10 element bases and with promoter spacer

**Figure 5.**
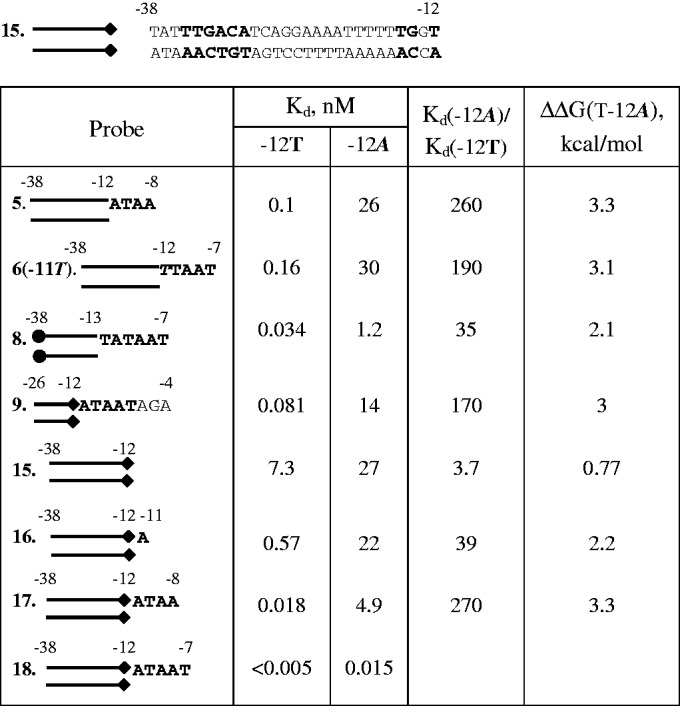
Effect of the T−12A substitution on RNAP binding to fork junction probes. Sequence of double-stranded parts of fork junction probes 15–18 is shown on the top of the figure.

A T at the −12 position is highly conserved among bacterial σ^70^-dependent promoters (23) and substitutions of −12T decrease transcription from many promoters (24,25). Substitution of a T/A_−__12_ base pair for an A/T base pair considerably decreases heparin resistance of RNAP complexes with fork junctions based on the *lac*UV5 promoter (32). In agreement with these data, we found that T−12A substitution decreased affinities of fork junction probes 5, 6(−11T) and 9 by 170- to 260-fold (Figure 5). Heparin resistance assay data and structural modeling indicate that both nt-strand T and t-strand A of the T/A_−__12_ base pair are recognized by the σ subunit (4,18). Consistently, we found that affinity of fork junction probe 8 bearing an unpaired T at −12 was less affected by the T-12A substitution than affinities of probes 5, 6(−11T) and 9 (Figure 5). In agreement with this result, a derivative of fork junction probe 5 lacking the template strand nucleotide at position −12 (probe 31) bound RNAP ∼10-fold weaker than probe 5 (Supplementary Figure S3).

The affinity of oligonucleotide probe 30 depends very slightly on the identity of the base at −12, whereas substitutions of −11A and −7T greatly affected the binding (Supplementary Table S1). Modest ∼5-fold effects of substitutions of consensus base at the −12 position on the affinity have been observed with similar oligonucleotides that contained additional bases upstream of the −12 position (3,6,17). We considered a possibility that RNAP can effectively interact with the T/A_−__12_ base pair in a ds probe truncated downstream at the −12 position. To strengthen specific binding of downstream DNA end, a TG motif of extended −10 element was incorporated in the probe (Figure 5). However, RNAP recognized the T/A_−__12_ base pair in resulting probe 15 poorly, as probe 15(−12A) bound RNAP only ∼4-fold weaker (Figure 5). Next, we determined affinities of fork junction derivatives of probes 15 and 15(−12A) bearing A_−__11_, A_−__11_TAA_−__8_ or A_−__11_TAAT_−__7_ stretches of the consensus −10 element bases. The data presented in Figure 5 show that these extensions considerably improve affinities of T−12 containing probes, similarly to what was observed with consensus probes 1, 2, 5 and 6 (Figure 1C). However, in the context of −12A probes, the extension from −12 to −11 results in a negligible change in affinity, whereas the extension to −8 only moderately improves the binding by ∼6-fold. In contrast, extension from −8 to −7 increases affinity by ∼300-fold in the context of 17(−12A) and 18(−12A) probes. Accordingly, the T−12A substitution strongly affects the binding of fork junction 17 (Figure 5).

The data show that RNAP efficiently recognizes the T/A_−__12_ base pair in fork junctions bearing long stretches of nt-strand −10 element bases, but the recognition is less effective in fork junction bearing the minimal A_−__11_ extension and is poor in ds and ss probes truncated at −12. In principle, recognition of the T/A_−__12_ base pair in probes 15 and 16 may be affected by fraying of probe termini (33). However, this effect cannot explain the large difference in the efficiency of recognition of unpaired −12T in the context of oligo 30 and fork junction 8. We propose that the position of the −12 bp in RNAP complex with probe 15 is not compatible with strong specific interaction of T/A_−__12_ with σ. However, tight RNAP binding both to the −10 element bases and to promoter spacer segment located between the −10 and −35 elements may be sufficient to change spacer or/and σ conformation and bring the −12 bp to a position that is optimal for the recognition of T/A_−__12_ and adjacent nt-strand −10 element bases. This implies that the recognition of T/A_−__12_ should be coupled with initiation of promoter melting.

### RNAP binding to promoter fragments bearing −10 element template strand bases

**Figure 6.**
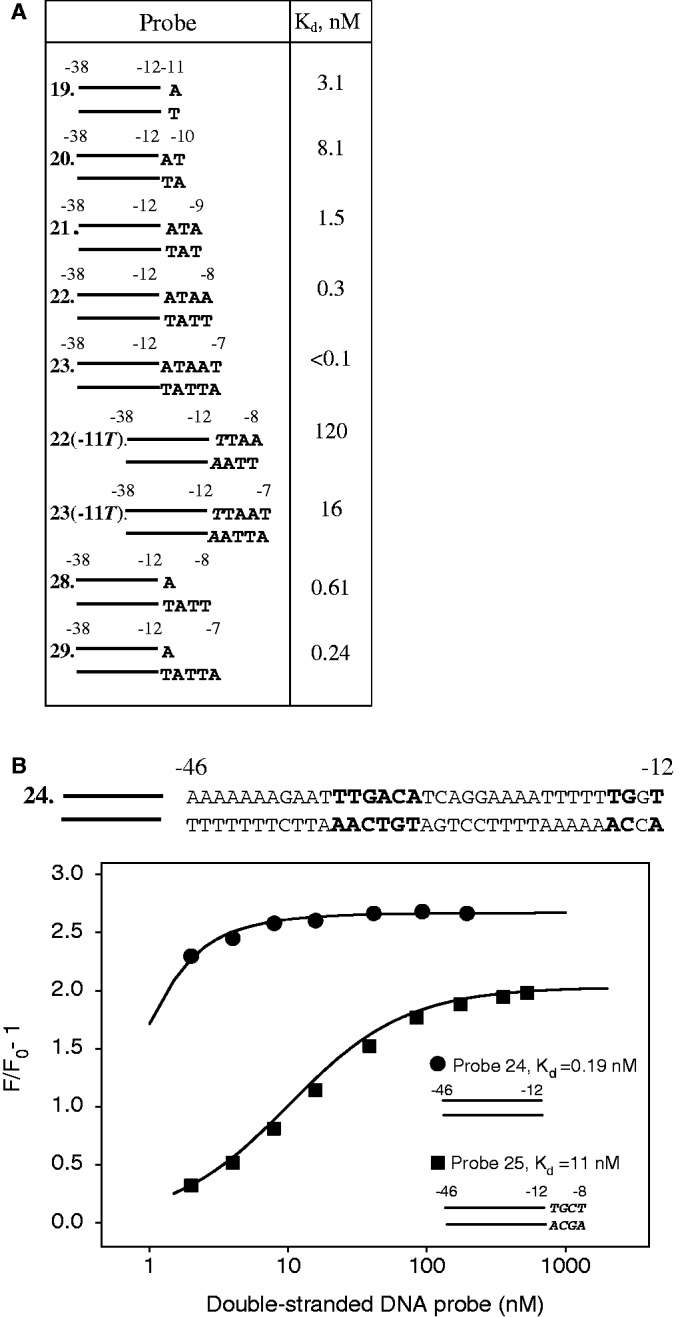
RNAP binding to promoter fragments bearing −10 element-template strand bases. (**A**) Calculated *K*_d_ values. The sequence of −38 to −12 segment of the probes corresponds to that of probe 1. (**B**) Inhibitory effect of ds segment bearing non-consensus −10 element bases (shown in italic) on RNAP binding to promoter fragment. The sequence of probe 24 is shown on the top of the panel.

Based on the aforementioned results, we created a set of ds and fork junction probes (probes 19–25, 27–29; Figure 6 and Supplementary Figure S1) bearing t-strand nucleotides downstream from the −12 position and measured affinities of these probes to RNAP. In the context of progressively extended ds probes 19–23, the introduction of the −10T/A bp resulted in inhibition of RNAP binding (Figure 6A), similarly to what was observed with fork junction probes. As expected, the A−11T substitution strongly affected affinity of ds probes. Probe 23(−11T) extended to −7 binds RNAP only 4-fold stronger than probe 1 bearing no nucleotides downstream from −12, whereas probe 22(−11T) with downstream end at −8 binds RNAP even weaker than probe 1 (*K*_d_ values are 16, 62 and 120 nM, respectively, Figures 1C and 6A). We further examined the effect of a ds segment bearing non-consensus −10 element bases in the context of probes 24 and 25 (Figure 6B) containing a sequence upstream of the −35 element which interacts effectively with the RNAP α subunit C-terminal domain (34) and a TG motif of extended −10 element. The data show that introduction of four non-consensus base pairs downstream from the −12 position in probe 24 leads to a ∼50-fold decrease in the affinity (Figure 6B). In contrast, fork junction derivatives of probe 24 containing either t-strand or nt-strand non-consensus bases bind RNAP stronger than the parent probe 24 (Supplementary Figure S4).

Feklistov and Darst (18) proposed that melting of the −11 and downstream positions allows the −12 position to move closer to a σ region 2.4 α helix, and that this movement is required for recognition of the −12 bp. This effect may at least in part account for the observed inhibition of the binding by mutations in positions −11/−8. Indeed, the approach of the −12 bp to σ region 2 should be constrained in RNAP complexes with ds probes 22(−11T) and 25 bearing non-consensus −10 element bases that likely remain paired. The large difference in affinities of probes 24 and 25 suggests that introduction of the non-consensus −10 element segment might affect not only the RNAP interaction with −12 position but also some other RNAP-promoter interactions in probe 25.

Previous investigations have revealed that RNAP binds to the t-strand segment of the transcription bubble considerably weaker than to the non-template segment (4,25). Consistently, we found that extensions of t-strand of probe 19 to the −8 and −7 position (respectively, fork junctions 28 and 29) conferred moderate (∼5- and 13-fold) improvements in affinities (Figure 6A).

## DISCUSSION

High-resolution structures of σ and RNAP bound to model promoter fragments have revealed that recognition of the −10 promoter consensus element is achieved through network of interactions between σ residues and nt-strand −10 element nucleotides extruded from the DNA double helix (18,19). In this work, we investigated whether these interactions are interdependent and, if so, whether such interdependency is essential for promoter binding. Accordingly, we measured how changing the identity of one −10 element nucleotide affects RNAP affinity to other −10 element nt-strand nucleotides in the context of promoter fragment DNA probes. The study required quantitative characterization of RNAP–DNA complexes with widely different stabilities, which is a technically challenging task because of RNAP propensity for non-specific DNA binding. Gralla and coworkers (4,29,32) studied the effects of substitutions in the −10 element on RNAP binding to DNA probes similar to those used in our work. However, the in-gel mobility retardation method they used allowed quantitative comparisons of probe affinities only within one order of magnitude range, which is insufficient for detection of effects related to cooperativity of RNAP interactions with the −10 element. We here relied on a highly sensitive molecular beacon RNAP assay that allowed quantitative characterization of RNAP–DNA complexes whose stabilities differed by as much as four orders of magnitude.

The main finding of our work is that specific RNAP interactions with nt-strand nucleotides of the −10 element are highly cooperative. The data also indicates that attaining a recognizable −10 element conformation is energetically costly. Accordingly, strong specific interaction with functionally most important and evolutionarily most conserved −10 promoter element bases occurs only when free energy gain resulting from the overall RNAP interaction with the −10 element exceeds a certain critical level. Such threshold-like behavior is a characteristic feature of various types of highly cooperative interactions (35,36). The threshold effect may help avoid unproductive RNAP interactions with occasional promoter-like DNA sequences, in particular when DNA melting is facilitated by negative DNA supercoiling, and thus improve the overall selectivity of promoter recognition.

RNAP interactions with the nt-strand −10 element nucleotides in progressively extended fork junctions (Figure 1) should reflect interactions that arise in RNAP-promoter complex on gradual expansion of the transcription bubble. An adenine at the −11 position is of special importance for nucleation of promoter melting (25,37–39). The data presented in Figure 1C demonstrate that specific interactions of −11A with RNAP in fork junctions with short A_−__11_ and A_−__11_T_−__12_ ss segments are considerably weaker than in fork junction bearing a single-stranded segment corresponding to complete −10 element. Accordingly, binding of the short ss fork junction segments to RNAP is also relatively weak (Figure 1C and D). The average free energy of a base pair breakage within an A/T rich −10 element-like sequence is ∼1 kcal/mol per bp (40), whereas initial nucleation of promoter melting can be much more energetically costly (40–42). This evaluation suggests that short bubbles around the −11A base, which may form because of thermal fluctuations (43,44), are unlikely to be stabilized by RNAP. In contrast, the ΔG gain resulting from RNAP interaction with ss stretch bearing −11 to −7 consensus −10 element bases (−7.1 kcal/mol, Figure 1D) should be sufficient to stabilize local melting. Thus, a first significantly stable melted intermediate promoter complex likely comprises unpaired −11 to −7 segment, at least in linear DNA templates. Overall, the results imply that cooperativity of promoter melting may be to a large degree accounted by the cooperativity of partial RNAP−10 element interactions. Our data also provide an explanation for the inhibitory effect of the −10 position (4), which is consistent with the proposed model of the −10 element recognition (18).

Our binding assays show that RNAP interacts with the T/A_−__12_ base pair in fork junctions much stronger than in a double-stranded promoter fragment 15 bearing no bases downstream of the −12 position (Figure 5). RNAP interaction with −12T is also weak in the context of single-stranded oligonucleotide probes. To explain these results, we propose that simultaneous RNAP binding to nt-strand bases of the −10 element and to double-stranded promoter spacer lead to a conformational change in promoter complex favoring specific recognition of the T/A_−__12_ base pair. Effective recognition of −12T in an ss DNA aptamer (28) suggests that some RNAP–aptamer interactions mimic RNAP contacts with promoter spacer. A sharp bend in DNA at the −16 position observed in the 6.5 Å resolution crystal structure of RNAP complex with a fork-junction promoter DNA (9) might be related to the hypothetical rearrangement improving the −12 bp recognition. Elucidation of this question may have to await high-resolution structures of various intermediates long the RP_o_ formation pathway.

The results obtained here also show that short ds segments bearing non-consensus −10 element bases decrease affinity of promoter fragments (Figure 6A and B). We note that ss stretches of non-consensus −10 element bases do not decrease the binding of fork junction probes (Supplementary Figure S4). This suggests us that duplex conformation of the −10 element can intrinsically impair promoter binding by constraining formation of RNAP–promoter contacts upstream from the −11 position. This explanation seems consistent with structural considerations indicating that RNAP interaction with unmelted −10 element segment should be weak and that unpairing of the −11 position and downstream −10 element bases should lead to strengthening of σ region 2 interactions with the −12T base (18). This effect should shift the equilibrium between duplex and melted conformations of the −10 element in the direction of melting, and thus may play a role in the DNA opening step.

## SUPPLEMENTARY DATA

Supplementary Data are available at NAR Online: Supplementary Table 1 and Supplementary Figures 1–4.

## FUNDING

National Institutes of Health [R01 GM64530 and R01 GM59295]; Molecular and Cell Biology Program grant from the Russian Academy of Sciences Presidium (to K.S.); ‘Scientific and scientific-pedagogical personnel of innovative Russia 2009–2013’ state contract 8475 (to V.M.). Funding for open access charge: ‘Scientific and scientific-pedagogical personnel of innovative Russia 2009–2013’ state contract 8475.

*Conflict of interest statement*. None declared.

## Supplementary Material

Supplementary Data
