# Supplementary material for: Cooperativity and interaction energy threshold effects in recognition of the −10 promoter element by bacterial RNA polymerase
Source: Nucleic Acids Res. 2013 Jun 14;41(15):7276–85. doi: 10.1093/nar/gkt541 (PMC3753650; doi:10.1093/nar/gkt541)
Supplement: Supplementary Data [file supp_41_15_7276__index.html]

Cooperativity and interaction energy threshold effects in recognition of the −10 promoter element by bacterial RNA polymerase — Cooperativity and interaction energy threshold effects in recognition of the −10 promoter element by bacterial RNA polymerase — Supplementary Data 

# Cooperativity and interaction energy threshold effects in recognition of the −10 promoter element by bacterial RNA polymerase

## 

files

**Files in this Data Supplement:**

- Supplementary Data - doc file
