# Supplementary material for: Cooperativity and interaction energy threshold effects in recognition of the −10 promoter element by bacterial RNA polymerase
Source: Nucleic Acids Res. 2013 Jun 14;41(15):7276–85. doi: 10.1093/nar/gkt541 (PMC3753650; doi:10.1093/nar/gkt541)
Supplement: Supplementary Data [file supp_gkt541_nar-01174-v-2013-File008.doc]

**Supplementary Data**

**Supplementary Table**

Consensus: **T A T A A T**

| Change  A  G  T  C | -12 -11 -10 -9 -8 -7 |
| --- | --- |
|  | 1.3 - 2.3 - - >50  1.1 >50 6.4 15 8.8 >50  - >50 - 13 10 -  0.9 >50 2 4.7 3.5 >50 |

**Table S1.** RNAP beacon assay for oligonucleotide probes with all substitutions in the -10 consensus. The probes are derivatives of oligonucleotide TATAATGGGTTCAT (probe 30 in Fig. S1) that bound RNAP with a Kd of 73 nM. Probe 30 corresponds to nt-strand segment of the T5 N25 promoter (positions the -12 to +2) except that the -4 and -6 bases are changed for G to disrupt a downstream -10 element like sequence. The affinity reduction caused by each substitution is shown. The consensus sequence is shown along the top. Probes 30(-8T), 30(-9T) and all probes bearing non-consensus bases at -11 or -7 generated very low fluorescence beacon signals. Dissociation constants of these probes were obtained from competition binding experiments using the consensus probe 30 as a reference, as described in (6). The Kd of other probes were determined using the titration assay. The Kd values presented are averages obtained from 2 individual experiments, the error is ±20%.

**Supplementary Figures**

-38 -12

**1.** TAT**TTGACA**TCAGGAAAATTTTTCTG**T**

ATA**AACTGT**AGTCCTTTTAAAAAGAC**A**

-38 -11 -38 -11

**2.** TAT**TTGACA**TCAGGAAAATTTTTCTG**TA 2(-11T).** TAT**TTGACA**TCAGGAAAATTTTTCTG**TT**

ATA**AACTGT**AGTCCTTTTAAAAAGAC**A**  ATA**AACTGT**AGTCCTTTTAAAAAGAC**A**

-38 -10 -38 -9

**3.** TAT**TTGACA**TCAGGAAAATTTTTCTG**TAT 4.** TAT**TTGACA**TCAGGAAAATTTTTCTG**TATA**

ATA**AACTGT**AGTCCTTTTAAAAAGAC**A** ATA**AACTGT**AGTCCTTTTAAAAAGAC**A**

-38 -12 -8 -38 -12 -7

**5.** TAT**TTGACA**TCAGGAAAATTTTTCTG**TATA 6.** TAT**TTGACA**TCAGGAAAATTTTTCTG**TATAAT**

ATA**AACTGT**AGTCCTTTTAAAAAGAC**A** ATA**AACTGT**AGTCCTTTTAAAAAGAC**A**

-38 -12 -8 -38 -12 -7

**7.** TAT**TTGCTT**TCAGGAAAATTTTTCTG**TATAA 8.** TAT**TTGCTT**TCAGGAAAATTTTTCTG**TATAAT**

ATA**AACGAA**AGTCCTTTTAAAAAGAC ATA**AACGAA**AGTCCTTTTAAAAAGAC

-26 -4

**9.** GGAAAATTTTT**TG**G**TATAAT**AGA

CCTTTTAAAAA**AC**C**A**

-38 -7 -38 -7

**10.** TAT**TTGACA**TCAGGAAAATTTTTCTG**TAGTTT 11.** TAT**TTGACA**TCAGGAAAATTTTTCTG**TTGTTT**

ATA**AACTGT**AGTCCTTTTAAAAAGAC**A** ATA**AACTGT**AGTCCTTTTAAAAAGAC**A**

-38 -12 -7 -38 -12 -7

**12.** TGG**TTGACT**TTTGCCAGATACTGAGG**CTGGCT 12(-7A).** TGG**TTGACT**TTTGCCAGATACTGAGG**CTGGCA**

ACC**AACTGA**AAACGGTCTATGACTCC**G** ACC**AACTGA**AAACGGTCTATGACTCC**G**

-38 -12 -8 -38 -12 -4

**13.** TGG**TTGACT**TTTGCCAGATACTGAGG**CTGGC**  **14.** TGG**TTGACT**TTTGCCAGATACTGAGG**CTGGCT**ATG

ACC**AACTGA**AAACGGTCTATGACTCC**G**  ACC**AACTGA**AAACGGTCTATGACTCC**G**

-38 -12 -38 -12

**15.** TAT**TTGACA**TCAGGAAAATTTTT**TG**G**T 15(-12A).** TAT**TTGACA**TCAGGAAAATTTTT**TG**G**A**

ATA**AACTGT**AGTCCTTTTAAAAA**AC**C**A** ATA**AACTGT**AGTCCTTTTAAAAA**AC**C**T**

-38 -11 -38 -11

**16.** TAT**TTGACA**TCAGGAAAATTTTT**TG**G**TA 16(-12A).** TAT**TTGACA**TCAGGAAAATTTTT**TG**G**AA**

ATA**AACTGT**AGTCCTTTTAAAAA**AC**C**A** ATA**AACTGT**AGTCCTTTTAAAAA**AC**C**T**

-38 -8 -38 -7

**17.** TAT**TTGACA**TCAGGAAAATTTTT**TG**G**TATAA 18.** TAT**TTGACA**TCAGGAAAATTTTT**TG**G**TATAAT**

ATA**AACTGT**AGTCCTTTTAAAAA**AC**C**A** ATA**AACTGT**AGTCCTTTTAAAAA**AC**C**A**

-38 -11 -38 -10

**19.** TAT**TTGACA**TCAGGAAAATTTTTCTG**TA 20.** TAT**TTGACA**TCAGGAAAATTTTTCTG**TAT**

ATA**AACTGT**AGTCCTTTTAAAAAGAC**AT** ATA**AACTGT**AGTCCTTTTAAAAAGAC**ATA**

-38 -9 -38 -8

**21.** TAT**TTGACA**TCAGGAAAATTTTTCTG**TATA 22.** TAT**TTGACA**TCAGGAAAATTTTTCTG**TATAA**

ATA**AACTGT**AGTCCTTTTAAAAAGAC**ATAT** ATA**AACTGT**AGTCCTTTTAAAAAGAC**ATATT**

-38 -12 -7

**23.** TAT**TTGACA**TCAGGAAAATTTTTCTG**TATAT**

ATA**AACTGT**AGTCCTTTTAAAAAGAC**ATATA**

-46 -12 -46 -12 -8

**24.** AAAAAAAGAAT**TTGACA**TCAGGAAAATTTTT**TG**G**T**  **25.** AAAAAAAGAAT**TTGACA**TCAGGAAAATTTTT**TG**G**TTGCT**

TTTTTTTCTTA**AACTGT**AGTCCTTTTAAAAA**AC**C**A** TTTTTTTCTTA**AACTGT**AGTCCTTTTAAAAA**AC**C**AACGA**

-46 -12 -8 -46 -12 -8

**26.** AAAAAAAGAAT**TTGACA**TCAGGAAAATTTTT**TG**G**TTGCT 27.** AAAAAAAGAAT**TTGACA**TCAGGAAAATTTTT**TG**G**T**

TTTTTTTCTTA**AACTGT**AGTCCTTTTAAAAA**AC**C**A**  TTTTTTTCTTA**AACTGT**AGTCCTTTTAAAAA**AC**C**AACGA**

-38 -12 -8 -38 -12 -7

**28.** TAT**TTGACA**TCAGGAAAATTTTTCTG**TA 29.** TAT**TTGACA**TCAGGAAAATTTTTCTG**TA**

ATA**AACTGT**AGTCCTTTTAAAAAGAC**ATAT** ATA**AACTGT**AGTCCTTTTAAAAAGAC**ATATTA**

-12 -7 +2-38 -12 -8

**30. TATAAT**GGGATTCAT **31.** TAT**TTGACA**TCAGGAAAATTTTTCTG**TATAA**

ATA**AACTGT**AGTCCTTTTAAAAAGAC

**Figure S1.** Structures of DNA probes used. The probe names used in the text are in red. The -10 and -35 promoter element sequences are highlighted in larger size font. Non-consensus -10 and -35 element bases are in pink.

**A**

-58 UP element -35 element -14

[-58/-14] GCTAAAATTTTTTTTAAAAGTAT**TTGACA**TCAGGAAAATTTTT**TG**

CGATTTTAAAAAAAATTTTCATA**AACTGT**AGTCCTTTTAAAAA**AC**

**B**

**Figure S2.** Competition binding assay for RNAP binding to fork junction probe 8. (**A**) Sequence of a reference competitor probe [-58/-14]*. (**B**) The panel shows the kinetics of decrease of beacon signal of RNAP beacon complex with fork junction 8 (black dots) resulted from competitive displacement of probe 8 by [-58/-14] upon addition of 2 nM [-58/-14] to the samples containing 1 nM *Ec* Eσ^70^ beacon and 2 nM probe 8 at 25 °C. An additional order of addition experiment in which probe 8 was added to the preformed (RNAP - [-58/-14]) complex (red dots) was carried out to verify that the competition assay reached equilibrium.

**A**

-38 -12 -8

**5.** TAT**TTGACA**TCAGGAAAATTTTTCTG**TATAA**

ATA**AACTGT**AGTCCTTTTAAAAAGAC**A**

-38 -13 -8

**31.** TAT**TTGACA**TCAGGAAAATTTTTCTG**TATAA**

ATA**AACTGT**AGTCCTTTTAAAAAGAC

**B**

**Figure S3.** Adenine at the -12 position of template strand stimulate binding of fork junction probe to RNAP. (**A**) Sequence of assayed fork junction probes 5 and 29. (**B**) The panel shows the kinetics of decrease of beacon signal of RNAP complexes with fork junction probes resulted from competitive displacement upon addition of 2 nM [-58/-14] to samples containing 1 nM RNAP beacon and 4 nM either 5 (black curve) or 29 (red curve) probe at 25 °C. A control order of addition experiment similar to that shown in Fig. S2B verified that competition assay for RNAP binding to probe 5 reached equilibrium (data not shown).

**Figure S4.** RNAP binding to double-stranded and fork junction probes bearing non-consensus -10 elements bases.
